# Drivers of Sinoatrial Node Automaticity in Zebrafish: Comparison With Mechanisms of Mammalian Pacemaker Function

**DOI:** 10.3389/fphys.2022.818122

**Published:** 2022-02-28

**Authors:** Matthew R. Stoyek, Eilidh A. MacDonald, Melissa Mantifel, Jonathan S. Baillie, Bailey M. Selig, Roger P. Croll, Frank M. Smith, T. Alexander Quinn

**Affiliations:** ^1^Department of Physiology and Biophysics, Dalhousie University, Halifax, NS, Canada; ^2^Institute of Cardiovascular and Medical Sciences, University of Glasgow, Glasgow, United Kingdom; ^3^Department of Medical Neuroscience, Dalhousie University, Halifax, NS, Canada; ^4^School of Biomedical Engineering, Dalhousie University, Halifax, NS, Canada

**Keywords:** heart rate, voltage clock, calcium clock, mechanics clock, autonomic nervous system, stretch, leading pacemaker site, blebbistatin

## Abstract

Cardiac excitation originates in the sinoatrial node (SAN), due to the automaticity of this distinct region of the heart. SAN automaticity is the result of a gradual depolarisation of the membrane potential in diastole, driven by a coupled system of transarcolemmal ion currents and intracellular Ca^2+^ cycling. The frequency of SAN excitation determines heart rate and is under the control of extra- and intracardiac (extrinsic and intrinsic) factors, including neural inputs and responses to tissue stretch. While the structure, function, and control of the SAN have been extensively studied in mammals, and some critical aspects have been shown to be similar in zebrafish, the specific drivers of zebrafish SAN automaticity and the response of its excitation to vagal nerve stimulation and mechanical preload remain incompletely understood. As the zebrafish represents an important alternative experimental model for the study of cardiac (patho-) physiology, we sought to determine its drivers of SAN automaticity and the response to nerve stimulation and baseline stretch. Using a pharmacological approach mirroring classic mammalian experiments, along with electrical stimulation of intact cardiac vagal nerves and the application of mechanical preload to the SAN, we demonstrate that the principal components of the coupled membrane- Ca^2+^ pacemaker system that drives automaticity in mammals are also active in the zebrafish, and that the effects of extra- and intracardiac control of heart rate seen in mammals are also present. Overall, these results, combined with previously published work, support the utility of the zebrafish as a novel experimental model for studies of SAN (patho-) physiological function.

## Introduction

In mammals, electrical excitation of the heart, which is responsible for its contraction and pumping action, originates in a distinct “pacemaker” region of the right atrium known as the sinoatrial node (SAN) (Gaskell, 1882; Keith and Flack, 1907). Cardiomyocytes in the SAN exhibit automaticity, due to a gradual depolarisation of their membrane potential (*V*_m_) in diastole (diastolic depolarisation, DD) that eventually reaches the threshold for action potential (AP) firing, whose frequency determines heart rate (HR) (Irisawa et al., 1993; Jansen et al., 2018; Quinn and Magder, 2021). While the relative importance of individual subcellular mechanisms of SAN automaticity have been intensely debated (DiFrancesco and Noble, 2012; Maltsev and Lakatta, 2012; Rosen et al., 2012), it is now generally accepted that pacemaker activity is driven by a robust system involving multiple overlapping contributors that maintain cardiac rhythm even when individual mechanisms are impeded (Lakatta and DiFrancesco, 2009; Noble et al., 2010).

### Mechanisms of Sinoatrial Node Automaticity

Mammalian studies have demonstrated that SAN automaticity is driven primarily by two systems of cellular mechanisms, commonly referred to as the membrane and Ca^2+^ “clocks” (consisting of transarcolemmal ionic currents and intracellular Ca^2+^ cycling, respectively). These two “clocks” form a system of tightly coupled oscillators (Lakatta et al., 2010) that are mutually entrained by intracellular regulatory mechanisms (MacDonald et al., 2020b). In the initial phase of diastole, DD in the SAN is caused by the opening of hyperpolarisation-activated cyclic nucleotide-gated (HCN) channels, which pass an inward cation current known as the “funny” current (*I*_f_) (DiFrancesco, 2010). As *V*_m_ becomes more positive, HCN channels begin to close, *I*_f_ decreases, and voltage-gated Ca^2+^ channels 3.1 (Ca_V_3.1) and 1.3 (Ca_V_1.3) begin to open, generating the depolarising transient (T-type) current (*I*_Ca,T_) and the Ca_V_1.3 component of the long-lasting (L-type) Ca^2+^ current (*I*_Ca,L_) that result in the continuation of DD (Mesirca et al., 2015). As diastole progresses, local release of Ca^2+^ from the sarcoplasmic reticulum (SR) *via* ryanodine receptors (RyR; known as “Ca^2+^ sparks”), occurring both spontaneously and being triggered by Ca_V_1.3 Ca^2+^ influx (Torrente et al., 2016), results in an increase in cytosolic Ca^2+^. A portion of this Ca^2+^ is returned to the SR by the sarco/endoplasmic reticulum Ca^2+^-ATPase (SERCA), with the remaining Ca^2+^ being extruded from the cell by the Na^+^—Ca^2+^ exchanger (with 3 Na^+^ ions entering the cell for every Ca^2+^ ion exiting), which generates an electrogenic, depolarising current (Lakatta et al., 2008). Ultimately, *V*_m_ reaches threshold for Ca_V_1.2 channel opening (∼ −40 mV), generating additional *I*_Ca,L_ (Mesirca et al., 2015), which is responsible for the upstroke of the SAN AP and results in SAN excitation. Repolarisation of *V*_m_ then occurs through opening of rapid and slow delayed rectifier K^+^ currents, which causes the re-activation of *I*_f_ and the beginning of a new phase of DD (Mangoni and Nargeot, 2008). Importantly, DD is not prevented by inwardly rectifying K^+^ channels (which stabilise and maintain the negative resting *V*_m_ of working cardiomyocytes), as those channels are minimally expressed or absent in SAN myocytes (Bartos et al., 2015).

### Control of Heart Rate

Sinoatrial node automaticity is strongly influenced by extracardiac and intracardiac factors, which alter transarcolemmal ion flux and intracellular Ca^2+^ handling, resulting in adaptation of HR to physiological changes. The principal extracardiac control of SAN function is *via* the central nervous system (CNS), acting through autonomic innervation of the heart (Ardell and Armour, 2016). Postganglionic sympathetic neurons project from the spinal cord and paravertebral ganglia directly to the cells of the heart (including in the SAN), where they release norepinephrine, which stimulates β-adrenergic receptors. The resulting G_αs_-coupled intracellular signalling increases HCN, L-type Ca^2+^, and delayed rectifier K^+^ channel activity, as well as SR Ca^2+^ release, which increases the slope of DD, and thus the frequency of SAN firing and HR (MacDonald et al., 2020b). In contrast, preganglionic parasympathetic neurons project from the brainstem to stimulate postganglionic intracardiac neurons, a portion of which project directly to SAN cells. There they release acetylcholine, which stimulates muscarinic receptors, resulting in G_αi/o_-coupled intracellular signalling (as well as G_βγ_-mediated activation of G-protein-coupled inward rectifying K^+^ channels), which reduces DD slope and thus HR (MacDonald et al., 2020b). While much of the neuronal input to the heart originates outside the organ, there is growing evidence for significant intracardiac, autoregulatory neurohormonal control of cardiac function (including SAN automaticity) by the intracardiac nervous system (IcNS) (Stoyek et al., 2021a). The IcNS is composed of a complex network of afferent, efferent, and interneurons (forming local circuits), which are embedded within the tissues of the heart. While the IcNS has traditionally been viewed as simply a “relay station” for extracardiac neuronal input from the CNS, it is now appreciated that the IcNS may in fact perform local processing and integration of CNS signals, as well as form intracardiac autoregulatory reflex loops (Armour, 2008). SAN automaticity is also affected by a host of additional extra- and intracardiac factors, including other neurotransmitters, neuropeptides, and circulating and locally released autocrine, paracrine, and endocrine factors. Interestingly, direct electrical stimulation of CNS inputs to the SAN, or pharmacologic activation of autonomic receptors on SAN cells not only affect HR, but also the spatial nature of SAN excitation, causing shifts in the intra-nodal site of initial excitation (“leading pacemaker site”) (Opthof, 1988; Boyett et al., 2000; Lang and Glukhov, 2021).

The predominant intracardiac factor governing SAN automaticity, however, is tissue stretch. In a wide variety of mammals (except for mice), stretch of the SAN results in a nearly instantaneous increase in HR (Quinn and Kohl, 2012; Quinn et al., 2011a). This occurs through acute feedback of the SAN’s mechanical state to its electrical activity *via* subcellular mechano-electric coupling (or mechano-electric feedback) mechanisms (Quinn et al., 2014; Quinn and Kohl, 2021). SAN mechano-sensitivity has long been known to affect HR, and the rapid response of the SAN to stretch is critical for its adaptation to constantly changing physiological conditions. In particular, the SAN stretch response allows for the beat-by-beat matching of cardiac output to physiological fluctuations in venous return and for preventing excessive bradycardia and over-distension of the right atrium by opposing the baroreceptor response when both venous return and arterial pressure are increased (Quinn, 2015; MacDonald and Quinn, 2021). Diastolic mechanical load (i.e., preload) of the SAN may also be important for the regularity of baseline rhythm. During atrial diastole, the right atrium and SAN are stretched by both right atrial filling and contraction of the ventricles, which pulls the atrio-ventricular valve-plane apically. Peak SAN stretch levels coincide with the period of DD, as *V*_m_ is moving toward the threshold for AP generation, such that subthreshold stretch-induced depolarisation may mechanically “prime” SAN cells for excitation, facilitating their steady firing. At the same time, stretch-induced depolarisation may help normalise electrical activity across the node, by the electrical entrainment of SAN cells. Combined, these effects may act to specifically enhance DD and “smooth out” differences in automaticity between cells across the SAN, thus stabilising cardiac rhythm (MacDonald and Quinn, 2021). Overall, this has led some to propose that the SAN stretch response represents a third primary driver of pacemaker activity, which may be considered the mechanics “clock” (Quinn and Kohl, 2012; MacDonald and Quinn, 2021).

### Zebrafish as a Model for Studies of Sinoatrial Node Function

The zebrafish is an increasingly important experimental model for the study of cardiac (patho-) physiology (Gut et al., 2017; Stoyek and Quinn, 2018; Echeazarra et al., 2021). The zebrafish has a fully sequenced genome, which may be altered using standard genetic techniques (Rafferty and Quinn, 2018; Stoyek et al., 2021b), and almost every cardiac gene has a human ortholog with analogous function (Howe et al., 2013). Functionally, the zebrafish heart has HR, AP morphologies, ion channels (Vornanen and Hassinen, 2016; Ravens, 2018), and Ca^2+^-handling proteins (Rayani et al., 2018; van Opbergen et al., 2018) that are generally more similar to human than are rodent characteristics. There are also differences between the zebrafish and human heart to be considered, including the zebrafish heart having two (rather than four) chambers, relatively low pressures (Hu et al., 2001), a degree of genetic redundancy due to genome duplications (Howe et al., 2013), and a greater dependency of contraction on transarcolemmal Ca^2+^ flux than SR Ca^2+^ release (Rayani et al., 2018; van Opbergen et al., 2018), which may limit its use for some applications.

The function and genetics of the zebrafish SAN is similar to that of human. In zebrafish, the SAN is found in a ring-like structure at the venous pole of the heart (the border between the venous sinus and the atrium), embedded within the leaflets of the sinoatrial valve (Arrenberg et al., 2010; Tessadori et al., 2012; Stoyek et al., 2015, 2016; Abu Nahia et al., 2021). Its development parallels that of the mammalian SAN, occurring from *isl1*-, *tbx*-, *bmp4*-, and *hcn4*-expressing cells under the control of *shox2* and facilitated by the suppression of *nkx2.5* through Wnt signalling (Burkhard et al., 2017; Martin and Waxman, 2021; Minhas et al., 2021). The first indication of the currents involved in zebrafish SAN automaticity came from the discovery of a mutation (*slow mo*) that caused a reduction in HR by affecting a hyperpolarisation-activated inward current with similar properties to *I*_f_ (Baker et al., 1997; Warren et al., 2001). More recently, the general involvement of the membrane and Ca^2+^ “clocks” in SAN automaticity were investigated using pharmacological interventions, which confirmed the role of *I*_f_ (block of HCN channels reduced HR by ∼65%) and demonstrated a role for intracellular Ca^2+^ cycling (block of SR Ca^2+^ release with ryanodine, combined with block of SR Ca^2+^ reuptake with thapsigargin reduced HR by ∼40%) (Marchant and Farrell, 2019). Yet, the potential involvement of other membrane and Ca^2+^ “clock” mechanisms (i.e., *I*_Ca_,_T_, *I*_Ca_,_L_) and the relative importance of these two pacemaker systems for SAN automaticity in the zebrafish has not been explored.

In terms of the extra- and intracardiac control of SAN automaticity, it has been shown that the response of HR to: (i) direct electrical stimulation of cardiac vagal nerves (Stoyek et al., 2016, 2018, 2021a); (ii) pharmacologic activation of autonomic receptors on SAN cells (Schwerte et al., 2006; Stoyek et al., 2016); (iii) local paracrine signalling (e.g., natriuretic peptides (Bendig et al., 2006; Becker et al., 2014; MacDonald et al., 2017); or (iv) tissue stretch (Werdich et al., 2012; MacDonald et al., 2017) is also similar to human and other mammals. Yet, aspects related to pacemaker shift with autonomic input and the importance of mechanical preload for the regularity of baseline rhythm have yet to be explored.

In the present study, we sought to determine the drivers of SAN automaticity in zebrafish, by mirroring classic pharmacological experiments used for their determination in mammals (and in particular rabbit), combined with confocal anatomical imaging and immunofluorescence assessment of the presence and distribution of key proteins for the membrane and Ca^2+^ “clocks” in the zebrafish SAN. This was followed by an investigation of open questions related to the extra- and intracardiac control of HR by vagal nerve stimulation and SAN stretch, specifically whether nerve stimulation leads to intra-nodal pacemaker shift (as seen in rabbits and other mammals) and whether mechanical preload stabilises cardiac rhythm (with matching rabbit experiments, for a mammalian comparison). Our results demonstrate that the principal components of the coupled membrane-Ca^2+^ oscillator system that drives automaticity in mammals are also active in the zebrafish SAN, and that the effects of extra- and intracardiac control of HR seen in mammals are also present in the zebrafish, supporting the utility of the zebrafish as an experimental model for studies of SAN pathophysiological function.

## Materials and Methods

### Ethical Approval

All experimental procedures were approved by the Dalhousie University Committee for Laboratory Animals and followed the guidelines of the Canadian Council on Animal Care. Details of experimental protocols have been reported following the Minimum Information about a Cardiac Electrophysiology Experiment (MICEE) reporting standard (Quinn et al., 2011b).

### Zebrafish Heart Isolation and Instrumentation

Hearts were isolated from wild-type (AB) adult zebrafish (6–12 months post fertilisation), as previously described (Stoyek et al., 2015, 2016, 2017, 2018; MacDonald et al., 2017). Zebrafish were euthanised with tricaine (1.5 mM MS-222; E10521, Sigma-Aldrich, Oakville, ON, Canada) in Tris-buffered (pH 7.4; BP152, Fisher Scientific, Ottawa, ON, Canada) room temperature tank water, until they showed no opercular respiration movements and lacked a locomotor response to fin pinch (∼2 min). They were then placed in a Sylgard-lined petri dish (DC 170, Dow Corning, Midland, MI, United States) filled with modified Krebs—Henseleit (KH) solution containing (in mM): 120 NaCl; 4.7 KCl; 26 NaHCO_3_; 1.4 NaH_2_PO_4_; 1.0 MgCl_2_; 1.8 CaCl_2_; 5.0 Glucose, with an osmolality of 290 ± 5 mOsm/kg and a pH of 7.40 ± 0.05 when bubbled with carbogen (95% O_2_, 5% CO_2_) at 28°C (additional details provided in Supplementary Table 1). A ventral midline incision was made through the body wall and pericardium to expose the heart. The heart was removed *via* incisions at the venous sinus (venous pole) and the ventral aorta (arterial pole) and placed in a separate Sylgard-lined petri dish and with 15 mL of the same modified KH solution bubbled with carbogen but maintained at 28.0 ± 0.5°C (physiological zebrafish temperature) with a temperature-controlled (TC-344C, Warner Instruments, Hamden, CT, United States) warmed platform (WP-16, Warner Instruments, Hamden, CT, United States). The heart was pinned in the dish with two needle electrodes (12 mm, 29 gauge; MLA1213, ADInstruments, Colorado Springs, CO, United States) at the arterial and venous poles of the heart, with a third reference electrode at the edge of bath. The electrodes were connected to an ECG amplifier (Animal Bio Amp, ADInstruments, Colorado Springs, CO, United States) and bath temperature was monitored using a thermocouple (T-type Pod, ADInstruments, Colorado Springs, CO, United States). Temperature and ECG signals were continuously recorded for the entire experiment at 2 kHz using a data acquisition device (PowerLab, ADInstruments, Colorado Springs, CO, United States) controlled by LabChart (ADInstruments, Colorado Springs, CO, United States). All experiments were performed in spontaneously beating hearts.

### Pharmacological Interventions

After a 10 min acclimatisation period, antagonists to the known principal components of the coupled membrane-Ca^2+^ oscillator pacemaker system in mammals were applied to the heart *via* the addition of stock solutions at the edge of the bath, followed by mixing *via* aspiration of solution with a transfer pipette. Concentrations and time of application were in the range previously used for rabbit, unless otherwise noted (additional details provided in Supplementary Table 2): (i) ivabradine hydrochloride for intracellular block of HCN channels (3 μM, 40 min) (Thollon et al., 1994, 2007; Bucchi et al., 2007; Yaniv et al., 2012, 2013, 2014); (ii) cesium chloride (CsCl) for extracellular block of HCN channels (3 mM, 7 min) (Noma et al., 1983; Dennis and Williams, 1986; Denyer and Brown, 1990; Leitch et al., 1995; Nikmaram et al., 1997; Zhang and Vassalle, 2000; Bogdanov et al., 2006; Thollon et al., 2007); (iii) nickel(II) chloride (NiCl_2_) for block of T-type Ca^2+^ channels (165 μM, 15 min; previous studies in rabbit used 40–100 μM (Hagiwara et al., 1988; Satoh, 1995), however previous work in zebrafish involving current measurements by patch clamp used a higher concentration (Nemtsas et al., 2010), which preliminary experiments demonstrated was more effective); (iv) ryanodine for block of RyR (1 μM, 15 min) (Hata et al., 1996; Satoh, 1997; Bogdanov et al., 2001, 2006; Vinogradova et al., 2002; Bucchi et al., 2003, 2007; Lancaster et al., 2004; Lyashkov et al., 2007); (v) BAPTA-AM for chelation of cytosolic Ca^2+^ (5 μM, 30 min exposure, followed by 30 min in modified KH alone to allow for de-esterification of the AM group) (Vinogradova et al., 2000; Bogdanov et al., 2006); and (vi) nifedipine for block of L-type Ca^2+^ channels (0.2 μM, 15 min) (Kawai et al., 1981; Senges et al., 1983; Satoh and Tsuchida, 1993; Masumiya et al., 1997; Budriesi et al., 1998; Bogdanov et al., 2006). Controls included: (i) 60 min with modified KH alone (time control); (ii) 30 min with 0.05% dimethyl sulfoxide (DMSO, followed by 30 min in modified KH alone for comparison with BAPTA-AM); and (iii) 15 min with 0.05% ethanol. HR (in beats per minute, bpm) was calculated from the R-waves of the ECG using custom routines in Matlab (MathWorks, Natick, MA, United States) and averaged over 3 min immediately before drug application and 3 min after the end of the drug incubation period. Stability of HR was assessed as the inter-beat variability of cycle length (CL) by calculating the root mean square of its successive differences (RMSSD_CL_) over each 3 min period.

### Zebrafish Atrial Isolation and Instrumentation

As in previous work (Stoyek et al., 2015, 2016, 2018; MacDonald et al., 2017), to expose the SAN, the ventricle was removed *via* resection at the atrioventricular junction, the atrium cut open, laid epicardial side down, and pinned flat. For functional experiments, a suction microelectrode, composed of a pulled glass capillary (1.00/0.58 mm outer/inner diameter; 1B100, World Precision Instruments, Sarasota, FL, United States) in a microelectrode holder (MPH6R10, World Precision Instruments, Sarasota, FL, United States) connected to the ECG amplifier, was positioned with a three-axis rack and pinion stage (62041, Edmond Optics, Barrington, IL, United States) at the edge of the atrium and suction applied with a 1 ml syringe to measure the local ECG, in combination with a 26-27 gauge polytetrafluoroethylene coated silver wire (AGT1510, World Precision Instruments, Sarasota, FL, United States) reference electrode positioned at the edge of the bath.

### Immunofluorescence

For immunofluorescence assessment of the presence and distribution of HCN channels and RyR in the zebrafish SAN, atria were isolated from zebrafish expressing eGFP under the myocyte-specific *myl7* promoter [*tg(myl7:eGFP)*] for visualisation of the cardiac musculature. As previously described (Stoyek et al., 2015, 2016, 2018), the atria were fixed overnight in 2% paraformaldehyde (PFA; RT-15710, Electron Microscopy Sciences, Hatfield, PA, United States) with 1% DMSO (BP231-1, Sigma-Aldrich, Oakville, ON, Canada) in phosphate-buffered saline (PBS, P3813, Sigma-Aldrich, Oakville, ON, Canada). The atria were then rinsed three times for 15 min each in PBS and transferred to a solution containing 0.1% Triton X-100 (PBS-T; T9284, Sigma-Aldrich, Oakville, ON, Canada) in PBS with primary antibodies for HCN4 channels (1:50; APC-052, Alomone Labs, Jerusalem, Israel) and RyR (1:100; MA3-916, Fisher Scientific) and incubated for 3–5 days with agitation at 4°C (additional details for antibodies and solutions provided in Supplementary Tables 3, 4). Tissues were rinsed three times for 15 min each in PBS-T and transferred to PBS-T containing the appropriate secondary antibody (1:300, AlexaFluor555 or AlexaFluor647; A-21429 or A-21236, Fisher Scientific – additional details provided in Supplementary Table 3) for 3–5 days with agitation at 4°C. Final rinsing was done in PBS and specimens were placed in Scale CUBIC-R1 clearing solution (Susaki et al., 2014; additional details provided in Supplementary Table 4) overnight at room temperature with gentle agitation. Atria were then mounted on glass slides in CUBIC-R1 for confocal microscopy.

Processed specimens were examined as whole-mounts using an LSM 510 confocal microscope (Carl Zeiss, Toronto, ON, Canada) with a plan-apochromat 10 × , 0.45 NA (SF25, Carl Zeiss) or 40 × , 0.80 NA (LCI Plan-Neofluar, Carl Zeiss) objective. Preparations were epi-illuminated with a 488 nm argon laser and 543 nm and 605 nm helium-neon lasers reflected by a 488/543/633 nm dichroic mirror (HFT 488/543/633; Carl Zeiss). Emitted fluorescence was collected using 480-520 nm and 530-585 nm band-pass filters and a 615 nm long-pass filter (Carl Zeiss). Confocal image Z-stacks were acquired and processed using Zeiss Zen2009 software from regions of interest surrounding immunoreactive tissues (omission of primary or secondary antibodies resulted in absence of immunoreactivity), which ranged from 50–350 μm in depth, including a region of 10–25 μm above and below the region of interest to ensure that all relevant structures were captured, while limiting issues of light scattering associated with deeper tissue scans. Figure plates were constructed from images processed with Photoshop (CS6, Adobe, Mountain View, CA, United States). Brightness and contrast of some images were adjusted to ensure panel-to-panel consistency in each figure.

### Voltage Optical Mapping and Microelectrode Recordings

Voltage optical mapping was performed to image excitation of the zebrafish SAN, as previously described (Stoyek et al., 2016, 2018). Isolated atria were left to acclimatise for 30 min, during the last 10 min of which a voltage-sensitive dye was added to the bath (10 μM, di-4-ANBDQPQ; provided by Leslie Loew, Richard D. Berlin Center for Cell Analysis and Modeling, University of Connecticut Health Center, Farmington, CT, United States). The atria were then washed with fresh modified KH solution containing the excitation-contraction uncoupler ( ± )-blebbistatin (10 μM; 13186, Cayman Chemical, Ann Arbor, MI, United States) to eliminate contraction-induced artifacts in optical recordings and left for an additional 30 min to allow blebbistatin to act. Preparations were epi-illuminated through a macro zoom microscope (MVX10, Olympus, Toronto, ON, Canada) with a mercury lamp (U-HGLGPS, Olympus, Toronto, ON, Canada) passed through a 630-650 nm band-pass filter (D640/20X, Chroma Technology, Bellows Falls, VT, United States) and reflected by a 685 nm dichroic mirror (FF85-Di02, Semrock, Rochester, NY, United States). Emitted fluorescence was collected with a 2 × , 0.50 NA objective (MV PLAPO 2XC, Olympus, Toronto, ON, Canada) at 4-6.3 × magnification through a 700 nm long-pass filter (ET700LP, Chroma Technology, Bellows Falls, VT, United States). Recordings were captured by a 128 × 128 pixel, 16-bit electron-multiplying charge-coupled device camera (Cascade 128+, Photometrics, Tucson, AZ, United States) at 511 frames/s using MultiRecorder software (provided by Johannes Schröder—Schetelig and Stefan Luther, Max Planck Institute for Dynamics and Self-Organization, Göttingen, Germany). To identify the site of initial SAN excitation, binary images were created for three successive cardiac cycles and the initiation site averaged using custom Matlab routines. An oval was overlaid on the image to fit the SAN and the binary images were mapped to a brightfield exemplar image of the SAN by rigid transformation using Fiji software (Schindelin et al., 2012).

To check for possible effects of blebbistatin on zebrafish electrophysiology (which has been shown in embryonic isolated hearts to have no effects; Jou et al. (2010), but has not been tested in adult hearts), intracellular microelectrode recordings of SAN *V*_m_ in isolated atrium (as well as recording of *V*_m_ in the atrium and ventricle of the whole heart) were acquired as previously described (Kopton et al., 2018), with and without application of blebbistatin. Microelectrodes made from borosilicate glass capillaries (1.0/0.5 mm outer/inner diameter, with internal filament; type BF/100/50/10, Sutter Instruments; Novato, AZ, United States) were pulled on a Brown/Flaming micropipette puller (Model P97, Sutter Instruments) to tip diameters resulting in a 40–60 MΩ resistance when filled with 3 M KCl. Electrodes were coupled to the headstage of an amplifier (Model 1600 Neuroprobe Amplifier, A/M Systems; Everett, WA, United States) operated in current clamp mode with an electrode holder (ESW-M10N, Warner Instruments, Hamden, CT, United States). Electrodes were advanced with a mechanical manipulator (MX/4, Narishige Group, Amityville, NY, United States) into the myocardium. Before cell penetration, the tip potential of the electrode was nulled using the bridge controls of the intracellular amplifier with the electrode tip in the bath. At the end of a recording, the microelectrode was withdrawn from the cell, the null potential was checked, and the previous data adjusted if necessary. *V*_m_ was taken as the difference between the potential measured in the bath with a silver/silver-chloride lead and the intracellular potential. Successful impalement was signalled by a sudden step of the electrode potential to a negative value. *V*_m_ was recorded at 10 kHz with the PowerLab data acquisition device controlled by LabChart (ADInstruments, Colorado Springs, CO, United States). From the *V*_m_ recordings, characteristics of the SAN, atrial, and ventricular cell AP were calculated, using custom routines in Matlab following previously described algorithms (Bucchi et al., 2007; Logantha et al., 2019). For the SAN, these included: (i) maximum diastolic potential (MDP), defined as the most negative *V*_m_ reached during AP repolarisation; (ii) slope of DD, defined as the change in *V*_m_ over the time between the point of MDP and the initiation of the AP, which was determined as the point at which the rate of change of *V*_m_ was greater than 500 mV/s; (iii) maximum rate of change of *V*_m_ during the upstroke (dV/dt_max_); (iv) maximum *V*_m_ during the AP; (v) AP amplitude, defined as the change in *V*_m_ between the initiation and peak of the AP; and (vi) AP duration at 50% (APD_50_) and 80% (APD_80_) repolarisation, defined as the time between the initiation of the AP and the restoration of *V*_m_ from peak to 50% or 80% of MDP. For the atrium and ventricle, resting *V*_m_ was calculated as the average value over the 25 ms immediately preceding the initiation of the AP.

### Cardiac Vagal Nerve Stimulation

Vagal nerve stimulation was performed as previously described (Stoyek et al., 2016, 2017, 2018). Approximately 1 mm of the rami of the cardiac branches of the left and right vagal nerves were exposed in the walls of the ducts of Cuvier. Bipolar silver wire electrodes were positioned on each with three-axis rack and pinion stages (MM-3, Narishige Group) and connected to constant current isolation units (PSIU6, Grass Instruments, Quincy, MA, United States) driven by a dual output square pulse stimulator (S88, Grass Instruments). The left or right vagal nerves were stimulated with trains of rectangular pulses (300 μA, 15 Hz, 0.5 ms pulse width, 10 s train duration). The site of initial SAN excitation was determined as baseline, during vagal nerve stimulation, and during vagal nerve stimulation after 15 min of exposure to the nicotinic receptor antagonist hexamethonium bromide (10 μM; H0879, Sigma-Aldrich, Oakville, ON, Canada).

### Rabbit Sinoatrial Node Isolation

Sinoatrial node were isolated from hearts of female rabbits (New Zealand White, 2.1 ± 0.2 kg), as previously described (MacDonald et al., 2020a). Rabbits were euthanised by ear vein injection of pentobarbital (140 mg/kg), followed by rapid heart excision, aortic cannulation, and Langendorff perfusion (20 mL/min) with modified KH solution containing (in mM): 120 NaCl; 4.7 KCl; 24 NaHCO_3_; 1.4 NaH_2_PO_4_; 1.0 MgCl_2_; 1.8 CaCl_2_; 10.0 glucose, with an osmolality of 300 ± 5 mOsm/kg and a pH of 7.40 ± 0.05 when bubbled with carbogen at 37 ± 0.5°C (additional details provided in Supplementary Table 1). The atria were removed by cutting along the atrio-ventricular valve plane and placed in a Sylgard-lined dish filled with the same modified KH solution at 37°C and bubbled with carbogen. The edge of each atrial appendage was pinned down with the bottom of the atrial appendages (which normally rest against the ventricles) face up, taking care to not strain tissue beyond its inherent dimensions. The endocardial side of the SAN was exposed by making incisions along the front surface of the superior and inferior *Venae cavae* (SVC and IVC). Excess fat and pericardial tissue were carefully removed from the underside of the preparation, without pulling or stretching the tissue. The rabbit SAN was then dissected along the medial edge of the crista terminalis and the interatrial septum.

### Sinoatrial Node Stretch

Zebrafish SAN stretch was performed as previously described (MacDonald et al., 2017). Two custom-made micro-sized hooks (∼100 μm in diameter), created from glass capillaries (2.00/1.12 mm outer/inner diameter; 1B200F, World Precision Instruments, Sarasota, FL, United States), were positioned opposing each other in the long-axis direction inside the SAN ring under an upright microscope (BX63, Olympus, Toronto, ON, Canada) with a 5 × , 0.15 NA objective (MPlanFL N, Olympus, Toronto, ON, Canada), such that the SAN tissue rested in the loops of the hooks without tissue puncture or damage. The hooks were held by microelectrode holders (MPH110, World Precision Instruments, Sarasota, FL, United States) that were coupled to three-axis hydraulic micromanipulators for fine positioning (MHW-103, Narishige International, East Meadow, NY, United States). One micromanipulator was mounted to a piezoelectric linear translator (P-621.1CD, Physik Instrumente, Auburn, AL, United States) for application of nm resolution computer-controlled movement (USB-6361, operated by custom routines developed in LabView, National Instruments, Austin, TX, United States), while the other remained stationary and was attached to an isometric force transducer (PY2 72-4491, Harvard Apparatus, Saint-Laurent, QC, Canada). Both units were fixed to stages on a rack and pinion track for coarse positioning (56798, Edmond Optics, Barrington, IL, United States). The ECG of the slack SAN was recorded, followed by application of ∼5 mg of tension to the tissue by slowly separating the hooks with the piezoelectric translator. HR and the RMSSD_CL_ were calculated from the ECG signal for the 30 s immediately before and the 30 s immediately after the application of mechanical preload using custom routines in Matlab.

Rabbit SAN stretch was performed as previously described (MacDonald et al., 2020a). Following SAN isolation, insect pins were woven through the cut edges of the SVC and IVC above and below the SAN tissue. A clip hanging from the isometric force transducer was attached to the SVC pin. A clip coupled to a computer-controlled linear DC-servomotor (LM 1247-02-01; FAULHABER MICROMO, Clearwater, FL, United States) was attached to and supported the IVC pin with bipolar electrodes at either end of the pin connected to the ECG amplifier. The entire SAN was immersed in a water-jacketed chamber containing the modified KH solution at 37°C and bubbled with carbogen. The ECG of the slack SAN was recorded, followed by application of ∼300 mg of tension to the tissue by slowly lowering the IVC pin with the DC-servomotor. As for the zebrafish, HR and RMSSD_CL_ were calculated from the ECG signal for the 30 s immediately before and the 30 s immediately after the application of mechanical preload using custom routines in Matlab.

### Statistical Analysis

Data are shown as mean ± standard error of the mean (SEM). Group means were compared either by paired or unpaired, two-tailed, Student’s *t*-tests or one-way, unpaired ANOVA and Tukey *post-hoc* tests, with a significance value of *p* < 0.05.

## Results

### Contribution of Membrane “Clock” Components to Zebrafish Sinoatrial Node Automaticity

**FIGURE 1:**
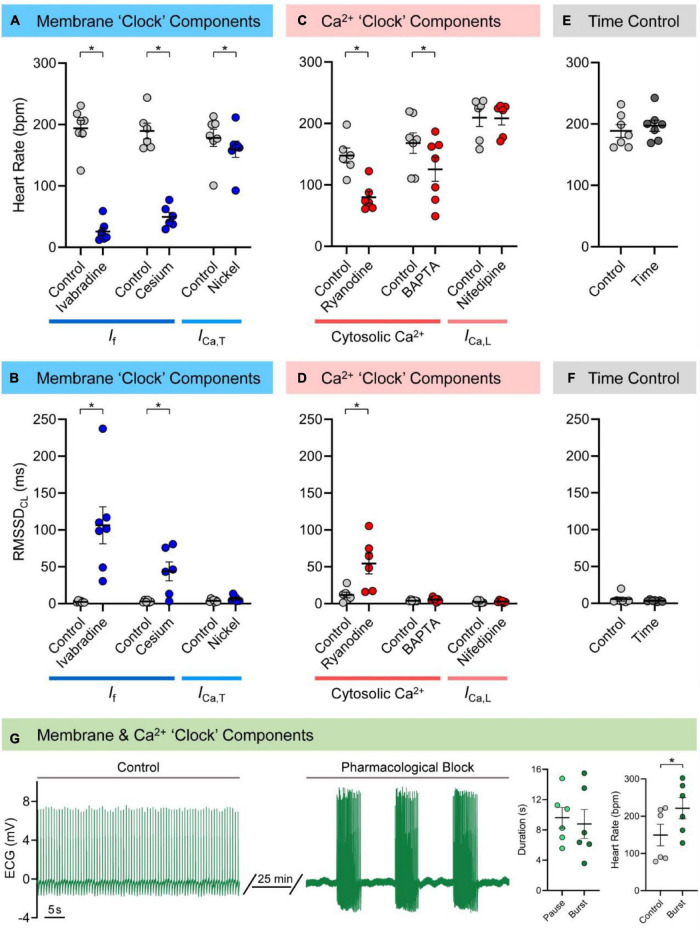
Mechanisms of zebrafish sinoatrial node (SAN) automaticity. Effects of pharmacological block of membrane (40 min of 3 μM ivabradine hydrochloride for intracellular block of “funny” current, *I*_f_, *n* = 7; 7 min of 3 mM cesium chloride for extracellular block of *I*_f_, *n* = 6; 15 min of 165 μM nickel(II) chloride for block of T-type Ca^2+^ current, *I*_Ca,T_, *n* = 7) or Ca^2+^ (15 min of 1 μM ryanodine for block of ryanodine receptors, *n* = 6; 30 min exposure of 5 μM BAPTA-AM, followed by 30 min to allow for de-esterification of the AM group, for chelation of cytosolic Ca^2+^, *n* = 7; 15 min of 0.2 μM nifedipine for block of L-type Ca^2+^ current, *I*_Ca,L_, *n* = 6) “clock” components, as well as matched time controls (60 min, *n* = 7) on heart rate **(A,C,E)** and the inter-beat variability of cycle length (measured as the root mean square of its successive differences, RMSSD_CL_; **B**,**D**,**F**), in the isolated zebrafish heart. Representative electrocardiogram (ECG) recording of zebrafish cardiac rhythm in control and after combined pharmacological block of membrane and Ca^2+^ “clock” components (3 mM cesium chloride, 165 μM nickel(II) chloride, and 1 μM ryanodine, *n* = 6) and measurements of the resulting SAN pause and burst duration and control and burst heart rate **(G)**. Data shown as mean ± SEM. Means compared by paired, two-tailed, Student’s *t*-tests; *p < 0.05.

Figures 1A,B show the effects of pharmacological block of membrane “clock” components on HR and RMSSD_CL_ in the isolated zebrafish heart (measured values are provided in Supplementary Table 5). Both *I*_f_ antagonists caused a decrease in HR (ivabradine: −168 ± 15 bpm or −86 ± 3%, *n* = 7, *p* < 0.001; cesium: −140 ± 15 bpm or −73 ± 4%, *n* = 6, *p* < 0.001) and an irregular inter-beat interval, so were associated with an increase in RMSSD_CL_ (ivabradine: +104 ± 25 ms, *p* = 0.006; cesium: +41 ± 13 ms, *p* = 0.023). The antagonist for *I*_Ca,T_ (nickel) also caused a decrease in HR (−18 ± 6 bpm or −10 ± 3%, *n* = 7, *p* = 0.016), but without a change in RMSSD_CL_ (*p* = 0.100). There was no change HR or RMSSD_CL_ in the time (Figures 1E,F; *n* = 7, *p* = 0.24, and *p* = 0.34) or vehicle (DMSO: *n* = 6, *p* = 0.30, and *p* = 0.66; ethanol: *n* = 6, *p* = 0.24, and *p* = 0.95) controls. There was no relationship between RMSSD_CL_ and the underlying HR for any of the pharmacologic agents used to block components of the membrane “clock.”

### Contribution of Ca^2+^ “Clock” Components to Zebrafish Sinoatrial Node Automaticity

Figures 1C,D show the effects of pharmacological block of Ca^2+^ “clock” components on HR and RMSSD_CL_ in the isolated zebrafish heart (measured values are provided in Supplementary Table 5). Block of RyR with ryanodine decreased HR (−68 ± 14 bpm or −45 ± 6%, *n* = 6, *p* = 0.005) and resulted in an irregular inter-beat interval, so was associated with an increase in RMSSD_CL_ (+43 ± 15 ms, *p* = 0.036). Exposure to BAPTA to chelate cytosolic Ca^2+^ also decreased HR (−43 ± 15 bpm or −26 ± 8%, *n* = 7, *p* = 0.029), but did not affect RMSSD_CL_ (*p* = 0.198). In contrast, block of *I*_Ca,L_ with nifedipine had no effect on HR (*p* = 0.813) or RMSSD_CL_ (*p* = 0.626). There was no relationship between RMSSD_CL_ and the underlying HR for any of the pharmacologic agents used to block components of the Ca^2+^ “clock.”

### Combined Block of Membrane and Ca^2+^ “Clock” Components

Figure 1G shows the effects of the combined pharmacological block of membrane and Ca^2+^ “clock” components (*I*_f_, *I*_Ca,T_, and RyR with cesium, nickel, and ryanodine) on SAN automaticity. The combined effect took between 10–25 min to stabilise, at which point there were long pauses in beating, followed by periods of a similar duration in which there were bursts of heart beats (9.6 ± 1.4 *vs.* 8.8 ± 1.9 s; *p* = 0.628). The HR of the bursts was greater than the baseline HR before pharmacological block (221 ± 28 *vs.* 149 ± 29 bpm; *p* = 0.048). Due to the nature of the heart rhythm after simultaneous pharmacological block of membrane and Ca^2+^ “clock” components (involving sinus pauses and bursts), RMSSD_CL_ was not measured in this case.

### Distribution of Hyperpolarisation-Activated Cyclic Nucleotide-Gated Channel-4 and Ryanodine Receptor in the Zebrafish Sinoatrial Node

**FIGURE 2:**
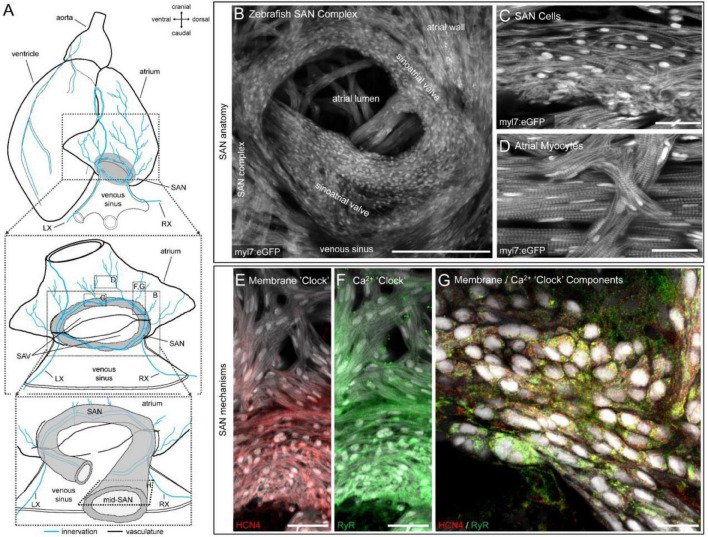
Structure and channel expression in the zebrafish sinoatrial node (SAN). Schematic of the zebrafish heart illustrating the location of the images in B-H **(A)**. Overview of the zebrafish SAN complex visualised by eGFP fluorescence in myl7-expressing cardiomyocytes **(B)**. Morphology of SAN cells **(C)** and atrial myocytes **(D)** in a 5 μm confocal *z*-projection. Immunofluorescence of hyperpolarisation-activated cyclic nucleotide-gated channel-4 (HCN4, red; **E**) and ryanodine receptor (RyR, green; **F**) expression in a 50 μm confocal *z*-projection through the SAN and proximal atrial myocardium. Cellular distribution of RyR and HCN4 in a single optical plane from approximately the mid-SAN **(G)**. Scale bars (in μm): **(B)**: 200 μm; **(C,D)**: 25 μm; **(E,F)**: 40 μm; **(G)**: 25 μm. LX, left cardiac branch of the vagal nerve; RX, right cardiac branch of the vagal nerves; SAV, sinoatrial valve.

Figure 2 shows the detailed structure of the zebrafish SAN (Figures 2A–D) and the expression of HCN4 channels and RyR, acquired by confocal imaging (Figures 2E–G). The genetically encoded eGFP, under the control of the myocyte-specific *myl7* promoter (*tg(myl7:eGFP)*), revealed a ring of cells at the venous pole of the heart completely surrounding the sinoatrial valve opening (Figure 2B). Cells within this ring were identified by a distinct spindle-shaped cellular morphology that varied with depth in the SAN and showed a reduced (but variable) expression of contractile apparatus (Figure 2C), distinct from myocytes in the atrial wall (Figure 2D). Immunofluorescence showed the highest HCN4 immunoreactivity in SAN cells, with minimal immunoreactivity observed beyond the proximal ends of atrial trabeculae connecting to the SAN (Figure 2E). Immunoreactivity of RyR was also highest in the SAN, but still observed at lower levels in the atrium (Figure 2F). In general, there was high cellular co-expression of HCN4 and RyR (Figure 2G).

### Effect of Vagal Nerve Stimulation on Leading Pacemaker Site of Zebrafish Sinoatrial Node

**FIGURE 3:**
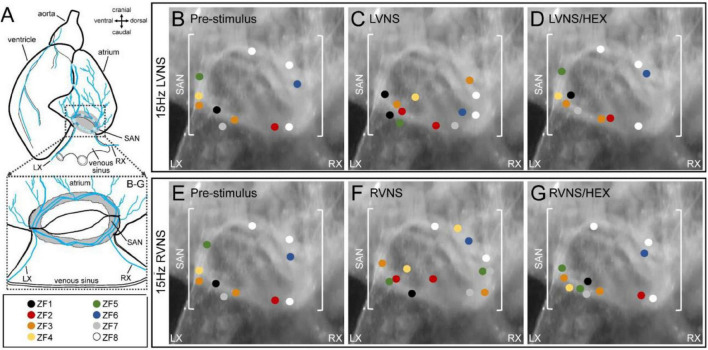
Effect of vagal nerve stimulation on leading pacemaker site of the zebrafish sinoatrial node (SAN). Schematic of the zebrafish isolated heart showing the location of the SAN and the intact left (LX) and right (RX) cardiac branches of the vagal nerve **(A)**. The leading pacemaker site(s) measured in individual SAN before nerve stimulation **(B,E)**, during left (LVNS, **C**) or right (RVNS, **F**) vagal nerve stimulation at 15 Hz, and during LVNS and RVNS after application of the nicotinic receptor blocker hexamethonium **(D,G)**, projected onto a representative SAN image, with individual subjects differentiated by colour (*n* = 8).

Figure 3 shows the initial site(s) of SAN excitation and the effect of left or right vagal nerve stimulation determined by voltage optical mapping. At baseline, the site of initial excitation varied between subjects, and was multi-focal in some cases (*n* = 2/8; Figures 3B,E). During left (LVNS; Figure 3C) or right (RVNS; Figure 3F) vagal nerve stimulation, there was a change in the location of initial excitation in each subject, with a greater incidence of multi-focal activity (left: *n* = 4/8; right: *n* = 6/8). After application of the nicotinic receptor blocker hexamethonium, the effect of vagal nerve stimulation on the initial site of SAN excitation was abolished.

### Effect of Blebbistatin on Electrophysiology of the Zebrafish Heart

**FIGURE 4:**
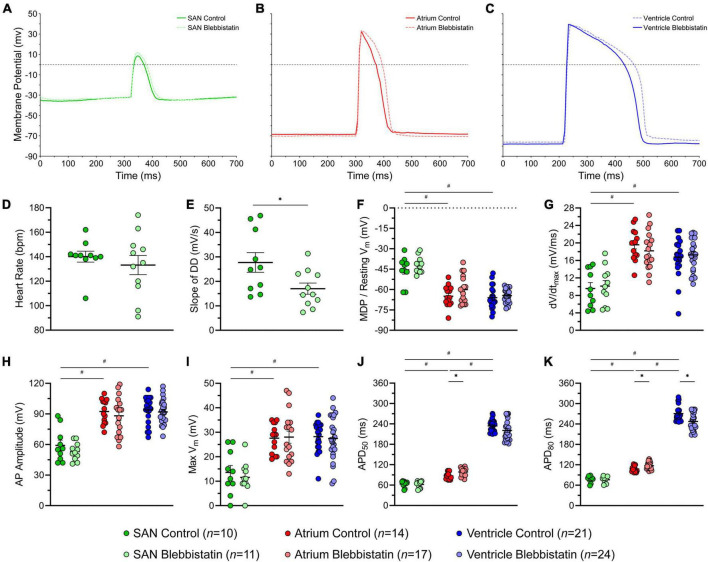
Effects of blebbistatin on the zebrafish sinoatrial node (SAN), atrial, and ventricular action potential (AP). Representative AP **(A–C)** and measured parameters **(D–K)** in the SAN (green), atrium (red), and ventricle (blue) with (dark) and without (light) blebbistatin (10 μM for 30 min). APD_50_ and APD_80_, AP duration at 50% and 80% repolarisation; DD, diastolic depolarisation; dV/dt_max_, maximum rate of change of membrane potential during the AP upstroke; MDP, maximum diastolic potential; *V*_m_, membrane potential. Data shown as mean ± SEM. SAN, atrium, and ventricle compared by unpaired, one-way ANOVA and Tukey *post hoc* tests; ^#^*p* < 0.05. Control and blebbistatin compared by unpaired, two-tailed, Student’s *t*-tests; *p < 0.05. *n* indicates number of impalements, which were acquired in *N* = 6 hearts.

Figure 4 shows AP characteristics from cells within the SAN, atrium, and ventricle with and without blebbistatin (measured values are provided in Supplementary Table 6). SAN cells had a less negative minimum *V*_m_ than atrial and ventricular cells, a lower dV/dt_max_, lower AP amplitude and max *V*_m_, and a shorter APD_50_ and APD_80_ (Figures 4F–K). Atrial cells had a shorter APD_50_ and APD_80_ than ventricular cells (Figures 4J,K). In the SAN, blebbistatin resulted in a reduction of the slope of DD (−39%, *p* = 0.028; Figure 4E), however the effect was not large enough to cause a reduction in HR (*p* = 0.463; although HR did become more variable, Figure 4D). In the atrium, blebbistatin resulted in an increase of APD_50_ and APD_80_ (+13% and +9%, *p* = 0.006 and *p* = 0.012; Figures 4J,K) and in the ventricle a decrease of APD_80_ (−9%, *p* = 0.003; Figure 4K). No other AP parameters were affected by blebbistatin (Figures 4F–K).

### Influence of Mechanical Preload on Heart Rate of the Zebrafish and Rabbit Isolated Sinoatrial Node

**FIGURE 5:**
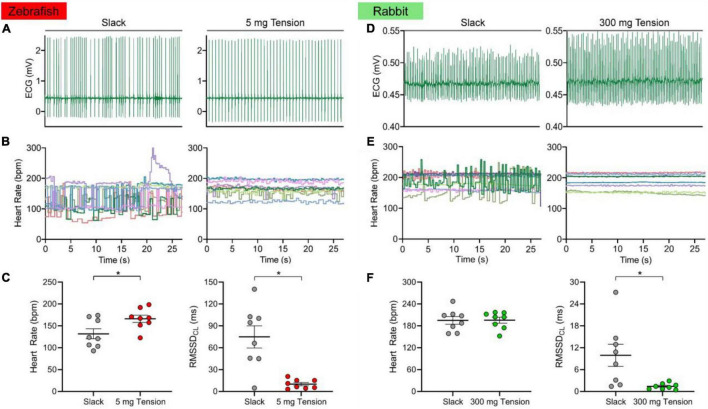
Effect of mechanical preload on zebrafish and rabbit sinoatrial node automaticity. Representative electrocardiogram (ECG) recording of zebrafish **(A)** and rabbit **(D)** sinoatrial node rhythm under unloaded (slack) or mechanically preloaded (5 mg or 300 mg) conditions. Heart rate over time of individual zebrafish (**B**, *n* = 8) and rabbit (**E**, *n* = 8) sinoatrial nodes when mechanically unloaded or preloaded. Average heart rate and the inter-beat variability of cycle length (measured as the root mean square of its successive differences, RMSSD_CL_) of the zebrafish **(C)** and rabbit **(F)** sinoatrial node when mechanically unloaded or preloaded. Data shown as mean ± SEM. Means compared by paired, two-tailed, Student’s *t*-tests; *p < 0.05.

Figure 5 shows the HR and RMSSD_CL_ of the slack or mechanically preloaded isolated zebrafish (*n* = 8) and rabbit (*n* = 8) SAN. When slack, the beating of the SAN in both species was irregular, as seen from the ECG traces (Figures 5A,D) and the beat-by-beat measurements of HR (Figures 5B,E). With the application of a static preload (zebrafish: 5.0 ± 0.7 mg; rabbit: 307.3 ± 49.3 mg), beating became much more rhythmic. This was associated with an increase in HR of the zebrafish (132 ± 12 *vs.* 166 ± 8 bpm; 30 ± 9%, *p* = 0.003; Figure 5C) but not the rabbit (195 ± 11 *vs.* 196 ± 8 bpm; *p* = 0.915; Figure 5F) SAN, while RMSSD_CL_ decreased in both species (74.9 ± 15.1 *vs.* 10.1 ± 2.2 ms; −78 ± 7%, *p* = 0.004 and 9.9 ± 3.0 *vs.* 1.4 ± 0.3 ms; −64 ± 14%, *p* = 0.031; Figures 5C,F).

## Discussion

**FIGURE 6:**
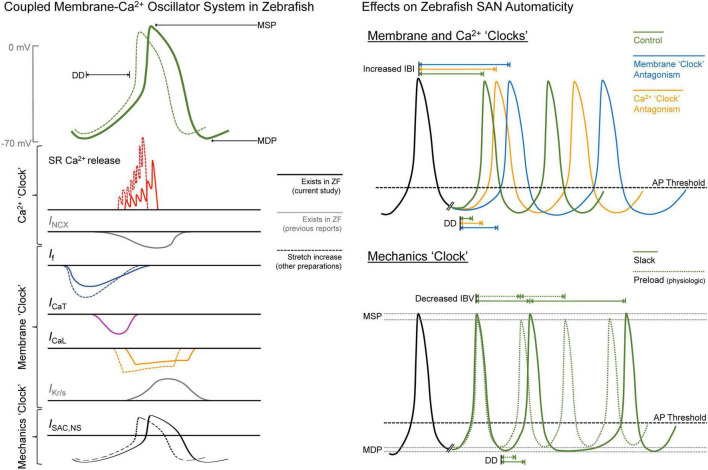
Summary of the drivers of zebrafish sinoatrial node (SAN) automaticity. AP, action potential; DD, diastolic depolarisation; *I*_Ca,L_, L-type Ca^2+^ current; *I*_Ca,T_, T-type Ca^2+^ current; IBI, inter-beat interval; IBV, inter-beat variation; *I*_f_, “funny” current; *I*_Kr/s_, rapid/slow delayed rectifier K^+^ current; *I*_NCX_, Na^+^-Ca^2+^ exchanger current; *I*_SAC,NS_, cation non-selective stretch-activated channels; MDP, maximum diastolic potential; MSP, maximum systolic potential; RyR, ryanodine receptors; SR, sarcoplasmic reticulum; ZF, zebrafish.

In this study, we explored open questions related to SAN automaticity in the zebrafish. Through pharmacological experiments based on those previously performed in mammals, we have demonstrated that the cellular mechanisms responsible for SAN automaticity are similar in the zebrafish (see summary in Figure 6). Further, we have shown that the principal extracardiac (e.g., autonomic nervous system) and intracardiac (e.g., mechanical preload) mechanisms controlling HR in mammals operate in a similar manner in the zebrafish SAN, resulting in a shift in the leading pacemaker site and a stabilisation of cardiac rhythm, respectively. Overall, these results add further support to previously published work suggesting the zebrafish as an experimental model for studies of SAN (patho-) physiological function.

### Cellular Mechanisms of Sinoatrial Node Automaticity

In our experiments, changes in HR with pharmacological antagonism of mechanisms known to be critical for SAN automaticity in mammals suggest that similar pacemaker mechanisms are present in the zebrafish heart (Figure 6). We found that block of *I*_f_ with ivabradine or cesium resulted in a large decrease in HR (−86 or −73%). Block of RyR with ryanodine, chelation of cytosolic Ca^2+^ with BAPTA, or block of *I*_Ca,T_ with nickel resulted in smaller decreases in HR (−45, −26, or −10%), while block of *I*_Ca,L_ with nifedipine had no effect (Figure 1). Previous experiments using rabbit isolated SAN tissue and cells also demonstrated decreases in HR with similar drug concentrations (a comparison, with relevant references, is provided in Table 1). While the decrease in HR with block of *I*_Ca,T_, RyR, or chelation of cytosolic Ca^2+^ in the current experiments were in the same range as previous reports in rabbit, the change in HR with *I*_f_ block was much larger, and we saw no change with block of *I*_Ca,L_, while others have shown a large decrease. This difference suggests that there is a different balance in the contribution of the various components of the membrane-Ca^2+^ oscillator system to SAN automaticity in the zebrafish compared to mammals, such that *I*_f_ plays a more prominent role, and the Ca_V_1.3 component of *I*_Ca,L_ is less important.

**TABLE 1:** Pharmacological interventions.

		Zebrafish		Rabbit		
Target	Antagonist	Concentration	Δ HR	Concentration	Δ HR	References
*I*_f_ (Intracellular)	Ivabradine	3 μM	−86%	3 – 5 μM	−13 – −21%	Thollon et al., 1994, 2007; Bucchi et al., 2007; Yaniv et al., 2012, 2013, 2014
*I*_f_ (Extracellular)	Cesium	3 mM	−73%	2 – 5 mM	−7 – −29%	Noma et al., 1983; Dennis and Williams, 1986; Denyer and Brown, 1990; Leitch et al., 1995; Nikmaram et al., 1997; Zhang and Vassalle, 2000; Bogdanov et al., 2006; Thollon et al., 2007
*I*_Ca,T_	Nickel	165 μM*	−10%	40 – 100 μM	−14 – −18%	Hagiwara et al., 1988; Satoh, 1995
*I*_Ca,L_	Nifedipine	0.2 μM	−	0.1 – 0.2 μM	−5 – −40%	Kawai et al., 1981; Senges et al., 1983; Satoh and Tsuchida, 1993; Masumiya et al., 1997; Budriesi et al., 1998; Bogdanov et al., 2006
RyR	Ryanodine	1 μM	−45%	1 – 3 μM	−14 – −50%	Hata et al., 1996; Satoh, 1997; Bogdanov et al., 2001, 2006; Vinogradova et al., 2002; Bucchi et al., 2003, 2007; Lancaster et al., 2004; Lyashkov et al., 2007
Cytosolic Ca^2+^	BAPTA-AM	5 μM	−26%	5 μM	−54 – −68%	Vinogradova et al., 2000; Bogdanov et al., 2006

Comparison of the effects of block of components of the coupled membrane-Ca^2+^ oscillator system on heart rate (HR) in zebrafish and rabbit isolated hearts. *Recent work in zebrafish used a higher concentration than previous rabbit studies (Nemtsas et al., 2010). I_Ca,L_, L-type Ca^2+^ current; I_Ca,T_, T-type Ca^2+^ current; I_f_, “funny” current; RyR, ryanodine receptors.

The apparent critical contribution of *I*_f_ to SAN automaticity in the zebrafish is supported by genetic studies in which a mutation causing a reduction in a hyperpolarisation-activated inward current with similar properties to *I*_f_ resulted in bradycardia (38% decrease in HR in embryos and 28% in surviving adults; Baker et al., 1997; Warren et al., 2001). More recent studies using a similar pharmacological approach as in our experiments have also shown that block of *I*_f_ (with 10 μM ivabradine or intraperitoneal injection of 4 μg/g zatebradine) causes a large decrease in zebrafish HR (60 – 65%; Lin et al., 2014; Marchant and Farrell, 2019).

Past studies also support the importance of RyR and intracellular Ca^2+^ cycling for zebrafish SAN automaticity shown in our study, as ryanodine (intraperitoneal injection of 50 ng/g), combined with thapsigargin (1.3 μg/g, to block SR Ca^2+^ reuptake) reduced HR by ∼40% (Marchant and Farrell, 2019). A similar result has been shown in another teleost (the trout), in which ryanodine (10 μM) and thapsigargin (1 μM) resulted in a 44% decrease in HR (Haverinen and Vornanen, 2007). In both zebrafish (Bovo et al., 2013) and trout (Llach et al., 2011), it has also been shown that Ca^2+^ sparks are present in atrial and ventricular myocytes. In zebrafish, however, their amplitude is smaller and duration greater compared to rabbit, due in part to a lower sensitivity of RyR to Ca^2+^ (Bovo et al., 2013). The lower sensitivity of RyR to Ca^2+^ in zebrafish may in part explain the smaller decrease in HR with *I*_Ca,L_ block seen in our experiments, as Ca^2+^ sparks triggered by Ca_V_1.3-mediated Ca^2+^ influx may play a less prominent role in SAN automaticity compared to mammals. Yet, while Ca_V_1.3-mediated Ca^2+^ sparks may not be present in zebrafish, and spontaneous Ca^2+^ sparks in zebrafish have characteristics differing from those in rabbit, RyR block still resulted in a similar decrease in HR (see Table 1). This could be due to a greater relative RyR expression, and thus total Ca^2+^ spark-induced Ca^2+^ efflux in SAN cells of zebrafish compared to rabbit or may be supported by the relatively higher Na^+^-Ca^2+^ exchanger activity seen in teleosts (as demonstrated in trout; Hove-Madsen et al., 2003) due to unique exchanger paralogs (Marshall et al., 2005).

The particular importance of *I*_f_ and (at least spontaneous) Ca^2+^ release *via* RyR to pacemaking in the zebrafish is also supported by the high expression of HCN and RyR protein shown in the SAN by immunofluorescence in our study (Figure 2), as well as the increase in the instability of cardiac rhythm with their block (Figure 1), as has been shown to also occur in rabbit (Yaniv et al., 2014). This instability may, however, partly be driven by the decrease in HR, which itself has been shown to increase HR variability (Rocchetti et al., 2000).

In regards to the contribution of *I*_Ca,T_ to SAN automaticity shown in our study, a robust *I*_Ca,T_ has been demonstrated in both the atrium and ventricle of the zebrafish heart (Nemtsas et al., 2010; Alday et al., 2014), so is presumably also present in the zebrafish SAN (T-type Ca^2+^ channels in the adult mammalian heart are in fact generally restricted to the SAN; Mesirca et al., 2015). Importantly, in studies in which HR was reduced with mutation of *I*_f_ channels it was shown that *I*_Ca,T_ was still present and unaffected (Baker et al., 1997), so would have been a contributor to the continued pacemaking in those animals.

It has been shown that compared to other regions of the heart, the zebrafish SAN overexpresses several signature mammalian pacemaker genes, including those encoding HCN and Ca^2+^- and K^+^-gated channels (von der Heyde et al., 2020; Minhas et al., 2021). In the context of SAN function, it has also been shown that knock down of the large-conductance Ca^2+^-activated K^+^ channel (K_Ca_1.1) in zebrafish, which is strongly expressed in the human SAN, results in sinus bradycardia (Pineda et al., 2021), so it may play an important role in zebrafish SAN automaticity through its contribution to repolarisation.

Interestingly, combined pharmacological antagonism of *I*_f_, *I*_Ca,T_, and RyR did not completely block beating but instead resulted in long SAN pauses interspersed by bursts of a higher frequency than the HR before pharmacological block. This suggests that when these primary molecular mechanisms of SAN automaticity in the zebrafish are inhibited, SAN excitation is prevented until other mechanisms overcome this inhibition and cause the heart to beat (for instance, mechano-electric coupling effects driven by SAN stretch, or large, sudden releases of Ca^2+^ from the SR due to severe Ca^2+^ overload), reflecting the overall complexity of the coupled membrane-Ca^2+^ oscillator pacemaker system. The mechanisms responsible for this finding warrant further investigation.

### Leading Pacemaker Shift With Vagal Nerve Stimulation

We found that the site of initial SAN excitation varied between individual zebrafish and was in some cases multi-focal. With vagal nerve stimulation there was a shift in the location of these sites and a greater incidence of multi-focal excitation. Effects differed with left *vs.* right nerve stimulation and were abolished by nicotinic receptor blockade, which prevents signal transmission from pre- to postganglionic neurons. It has been shown previously in zebrafish (Stoyek et al., 2016), as well as in carp (Saito and Tenma, 1976), that left or right vagal nerve stimulation leads to bradycardia (due to parasympathetic activation), followed by a post stimulation tachycardia (due to sympathetic activation), as in mammals. Simultaneous left-right nerve stimulation results in a summative HR effect, as the left and right vagal nerves innervate distinct regions of the SAN, proximal to their entry into the heart (Stoyek et al., 2015). In fact, approximately 90% of all intracardiac neurons in the zebrafish heart are located within the SAN region, with terminals concentrated near intracardiac ganglia adjacent to cells expressing HCN4 (the principal HCN isoform contributing to SAN automaticity in zebrafish and mammals; Stoyek et al., 2015), as well as adrenergic and cholinergic receptors (Stoyek et al., 2015). It has also been demonstrated that excessive vagal nerve stimulation can silence the SAN (presumably due to activation of the acetylcholine-activated K^+^ current, which is present in zebrafish; Nemtsas et al., 2010), causing a shift in the origin of heart excitation toward the atrioventricular node (Stoyek et al., 2016).

A shift in the leading pacemaker site with electrical stimulation of extracardiac inputs or during pharmacologic activation of autonomic receptors has been demonstrated in a variety of mammals (Opthof, 1988; Boyett et al., 2000; Lang and Glukhov, 2021), including rabbit (West, 1955; Toda and West, 1967; Bouman et al., 1968; Toda, 1968; Toda and Shimamoto, 1968; Spear et al., 1979; Mackaay et al., 1980; Shibata et al., 2001; Lang et al., 2011). In general, sympathetic stimulation tends to shift the leading pacemaker site superiorly, while parasympathetic simulation results in an inferior shift, with the distance of shift scaling with the associated change in HR. These shifts have been attributed primarily to regional heterogeneity across the SAN, in terms of the balance of pacemaker currents in SAN cells and their responsiveness to autonomic stimulation, either due to differences in local innervation or receptor density (Opthof, 1988; Boyett et al., 2000; Lang and Glukhov, 2021). In the current study, the multi-focal nature of the initial site of SAN excitation, combined with its change during vagal nerve stimulation, suggest that there may be a similar heterogeneity in the zebrafish SAN.

The diameter of the SAN in mature zebrafish (6–12 months post fertilisation) has been reported to range between 200–600 μm, depending on subject-specific size and age (Stoyek et al., 2015, 2016; MacDonald et al., 2017). Based on nuclei counts from confocal z-projections of the SAN, the number of pacemaker cells is estimated to be in the low-to-mid thousands. In the current study we observed morphological differences in SAN cells across the node. In terms of functional heterogeneity, microelectrode recordings of SAN cell AP have shown variation in measured parameters (e.g., slope of DD, MDP, dV/dt_max_, AP amplitude, APD), however findings are too preliminary to make strong conclusions about the spatial heterogeneity of electrical properties. In terms of the currents underlying pacemaker activity, to our knowledge no study has investigated differences in current densities or protein or gene expression across the SAN, which is an interesting topic for future experimental work. Non-uniform local innervation of the zebrafish SAN has already been demonstrated (Stoyek et al., 2015), such that the distinct regions of SAN innervation from the left and right vagal nerves may explain the differences in pacemaker shift observed during their individual stimulation. The potential for heterogeneity of pacemaker currents and autonomic receptors across the SAN, however, remains to be investigated, although it appears there are regional differences in SAN automaticity from the earliest stages of zebrafish development (Arrenberg et al., 2010).

### Importance of Mechanical Preload to Pacemaker Function

We found that HR of the slack zebrafish SAN in isolated atria was irregular, and that rhythm was stabilised by static stretch of the SAN to a mechanical preload (Figure 6). This finding was corroborated in rabbit isolated SAN. Sustained stretch of the SAN has long been known to cause an acute increase in HR (Quinn et al., 2011a; Quinn and Kohl, 2012), including in rabbit (Quinn and Kohl, 2016; MacDonald et al., 2020a) and zebrafish (MacDonald et al., 2017). This chronotropic effect is thought to occur through activation of cation non-selective stretch-activated channels, and perhaps also through mechano-sensitive components of the coupled membrane-Ca^2+^ oscillator pacemaker system (Quinn and Kohl, 2012, 2021). The critical nature of a “minimal” diastolic tension for the generation and stabilisation of rhythmic SAN excitation was first suggested by experiments in cat (Lange et al., 1966), dog (Brooks et al., 1966) and rabbit (Ushiyama and Brooks, 1977) isolated SAN, in which stretch either restored regular rhythm in slack SAN with irregular activity or resulted in spontaneous excitation in previously quiescent SAN. In such cases, it is apparent that instability or SAN quiescence is due to the failure of other pacemaker mechanisms to sustain DD, which is restored by a sufficient mechanical preload, along with a positive shift in the maximum diastolic potential towards AP threshold. In fact, the importance of an adequate preload for SAN pacemaking may be present from the very first heartbeat during embryonic development, as fluid pressure build-up in the quiescent cardiac tube appears to be a pre-requirement for the initiation of spontaneous cardiac excitation during ontogenesis (Rajala et al., 1976, 1977; Chiou et al., 2016). Our current experiments demonstrate that the critical nature of a mechanical preload for rhythmic SAN firing (MacDonald and Quinn, 2021) applies to both zebrafish and rabbit hearts.

### The Use of Blebbistatin With the Zebrafish Heart

We found that the application of blebbistatin to the isolated zebrafish heart resulted in some changes to its electrophysiology. While HR and most measured AP characteristics were unaffected, blebbistatin caused a decrease in the slope of DD in the SAN, an increase in APD in the atrium, and a decrease of APD in the ventricle. Reports of the effects of blebbistatin on cardiac electrophysiology in mammals have been variable. In isolated rabbit hearts, right ventricular and atrial tissue, and in rat ventricular myocytes, it was first shown that blebbistatin has no effect on SAN activity or on cardiomyocyte APD (Fedorov et al., 2007). Since that time, studies using rabbit hearts have confirmed a lack of an effect of blebbistatin on APD, while later studies using rabbit and pig hearts have demonstrated an increase (Swift et al., 2021). The increase in APD that we observed in the zebrafish atrium agrees with these later findings. It is unclear whether this increase in APD is due to non-specific interactions of blebbistatin with cardiac ion channels and/or transporters or is instead a secondary effect of the loss of contraction caused by excitation-contraction uncoupling, for instance due to a loss of mechano-electric coupling effects or to a decrease in sarcolemmal ATP-sensitive K^+^ channel current caused by a decrease in metabolic demand (Swift et al., 2021). The other electrophysiological findings in our study (decreased DD slope in the SAN and increased APD in the ventricle) suggest there may be direct effects of blebbistatin on ion channels and/or transporters, warranting further investigations to determine the specific mechanism(s) involved. It is worth noting that blebbistatin may also affect other aspects of cardiac electrophysiology (*e.g.*, conduction, refractoriness, APD restitution, arrhythmia threshold), as well as Ca^2+^ handling (*e.g.*, cytosolic Ca^2+^ levels, Ca^2+^ transient amplitude and duration), important for the function of the SAN and working myocardium (Swift et al., 2021), which deserve consideration in future studies using zebrafish.

### Experimental Limitations

The principal limitation of our approach is the use of pharmacological interventions to determine the relative importance of the various components of the coupled membrane-Ca^2+^ oscillator pacemaker system. While our results show in principle which components are relevant for SAN automaticity in the zebrafish, the differences in HR change with *I*_f_ and *I*_Ca,L_ block compared to rabbit could reflect differences in the level or specificity of block, or the presence of unappreciated channel paralogs in zebrafish not found in mammals (Jackson et al., 2007), rather than reflecting a relative difference in their contribution. This effect could be compounded by the use of drug concentrations previously used in mammals, but generally not corroborated in zebrafish. Future studies involving genetic manipulations of the relevant genes may help further define the particular importance of each of the mechanisms contributing to SAN automaticity in the zebrafish.

When considering the regional excitation of the SAN, its shift with vagal nerve stimulation, or the effects of mechanical preload, it is important to consider that the zebrafish SAN is a ring-shaped structure, as opposed to the sheet-like structure of the mammalian SAN, and that the vagal nerve carries both parasympathetic and sympathetic inputs (Stoyek et al., 2015). This will affect source-sink relations important for SAN excitation as well as the regional distribution of applied stretch and resulting tissue tension.

Finally, in the zebrafish experiments of the current study sex of the animals was not considered, while in the rabbit experiments SAN were isolated only from female animals. Although we are not aware of any reported differences in male *versus* female zebrafish or rabbit SAN, sex differences in the function and molecular mechanisms of the SAN have been described in rat (Doris et al., 2019), so should be considered in future studies.

## Conclusion

Here we have demonstrated that the cellular mechanisms responsible for SAN automaticity in zebrafish, as well as critical aspects of the control of SAN automaticity by the autonomic nervous system and tissue stretch, are in general similar to those in mammalian hearts (summarised in Figure 6). These findings accord with previous work considering other aspects of zebrafish SAN structure, genetics, composition, and function, thus further supporting the use of zebrafish as an experimental model for studies relating to SAN (patho-) physiology. The zebrafish has several other advantages as an experimental model for cardiac investigations, including its well-defined genome, ease of genetic manipulation, high fecundity, and relatively fast generation time, as well as the external development of nearly optically transparent embryos and relatively thin cardiac tissue that allow for cell-level measurement and manipulation of structure and function *in vivo* during development and *in situ* in the isolated heart. Combined, these aspects make the zebrafish particularly useful for genetic studies and studies involving advanced light-based techniques, such as live fluorescence imaging and optogenetic manipulations (Baillie et al., 2021). Its use therefore represents an exciting opportunity for innovative, cell-specific investigations into unexplored aspects of (patho-)physiological SAN function and control. In fact, the zebrafish has recently been utilised to explore the impact of ageing on the function and control of the SAN (Stoyek et al., 2018) and to model age-related sinus arrest and sick sinus syndrome (Yan et al., 2020). Similar studies in the future hold the promise of providing further clinically relevant novel insight into the essential contribution of the SAN to cardiac function.

## Data Availability Statement

The raw data supporting the conclusions of this article will be made available by the authors, upon reasonable request.

## Ethics Statement

All experimental procedures were approved and reviewed by the Dalhousie University Committee for Laboratory Animals and followed the guidelines of the Canadian Council on Animal Care.

## Author Contributions

MS, EM, JB, RC, FS, and TQ: conception and design of experiments. MS, EM, MM, JB, and BS: data collection. MS, EM, MM, JB, BS, and TQ: data analysis. MS, EM, and TQ: drafting of manuscript. All authors approved the final manuscript.

## Conflict of Interest

The authors declare that the research was conducted in the absence of any commercial or financial relationships that could be construed as a potential conflict of interest.

## Publisher’s Note

All claims expressed in this article are solely those of the authors and do not necessarily represent those of their affiliated organizations, or those of the publisher, the editors and the reviewers. Any product that may be evaluated in this article, or claim that may be made by its manufacturer, is not guaranteed or endorsed by the publisher.
