# Supplementary material for: Drivers of Sinoatrial Node Automaticity in Zebrafish: Comparison With Mechanisms of Mammalian Pacemaker Function
Source: Front Physiol. 2022 Feb 28;13:818122. doi: 10.3389/fphys.2022.818122 (PMC8919049; doi:10.3389/fphys.2022.818122)
Supplement: Supplementary file 1 [file Table_1.pdf]

Supplementary Table 1. Composition of zebrafish and rabbit saline solutions.

Substance / Parameter	Zebrafish	Rabbit	Vendor (Catalogue)
NaCl	120 mM	120 mM	Sigma-Aldrich (S5886)
KCl	4.7 mM	4.7 mM	Sigma-Aldrich (P5405)
$NaHCO_3$	26 mM	24 mM	Sigma-Aldrich (S6297)
NaH_2PO_4	1.4 mM	1.4 mM	Sigma-Aldrich (S0751)
$MgCl_2$	1.0 mM	1.0 mM	Sigma-Aldrich (63020)
$CaCl_2$	1.8 mM	1.8 mM	Sigma-Aldrich (21114)
Glucose	5.0 mM	10 mM	Sigma-Aldrich (G8270)
Osmolality	290±5 mOsm/kg	300±5 mOsm/kg	-
Bubbling	95% O_2 / 5% CO_2	95% O_2 / 5% CO_2	Linde Canada (MM OXCD5C)
pH	7.4	7.4	-
Temperature (°C)	28.0±0.5	37.0±0.5	-
