# Supplementary material for: Drivers of Sinoatrial Node Automaticity in Zebrafish: Comparison With Mechanisms of Mammalian Pacemaker Function
Source: Front Physiol. 2022 Feb 28;13:818122. doi: 10.3389/fphys.2022.818122 (PMC8919049; doi:10.3389/fphys.2022.818122)
Supplement: Supplementary file 2 [file Table_2.pdf]

Supplementary Table 2. Antagonists for block of coupled membrane-Ca^{2+} oscillator pacemaker system.

Target	Antagonist	Solvent	[Stock] (mM)	Stock Volume (µL)	[Final] (µM)	Time (min)	Vendor (Catalogue)	References
HCN Channels (Intracellular)	Ivabradine Hydrochloride	H_2O	1	45	3	40	Sigma-Aldrich (SML0281)	Bucchi et al., 2007; Thollon et al., 1994, 2007; Yaniv et al., 2012, 2013, 2014
HCN Channels (Extracellular)	Cesium Chloride (CsCl)	H_2O	300	150	3000	7	Sigma-Aldrich (C4036)	Bogdanov et al., 2006; Dennis and Williams, 1986; Denyer and Brown, 1990; Leitch et al., 1995; Nikmaram et al., 1997; Noma et al., 1983; Thollon et al., 2007; Zhang and Vassalle, 2000
T-type Ca^{2+} Channels	Nickel(II) Chlroide ($NiCl_2$)	H_2O	10	248	165*	15	Sigma-Aldrich (339350)	Hagiwara et al., 1988; Satoh, 1995
Ryanodine Receptors	Ryanodine	Ethanol	1	5	1	15	AbCam (ab120083)	Bogdanov et al, 2001, 2006; Bucchi et al., 2003, 2007; Hata et al., 1996; Lancaster et al., 2004; Lyashkov et al., 2007; Satoh, 1997; Vinogradova et al, 2002
Cytosolic Ca^{2+}	BAPTA-AM	DMSO	10	7.5	5	30**	Sigma-Aldrich (A1076)	Bogdanov et al., 2006; Vinogradova et al., 2000
L-type Ca^{2+} Channels	Nifedipine	DMSO	0.3	10	0.2	15	AbCam (ab120135)	Bogdanov et al., 2006; Budriesi, et al. 1998; Kawai et al., 1981; Masumiyaet al., 1997; Satoh and Tsuchida, 1993; Senges et al., 1983
Controls	Time (H_2O)	-	-	-	-	60	-	-
	DMSO	-	100%	7.5	0.05%	30***	Sigma-Aldrich (D2650)	-
	Ethanol	-	100%	7.5	0.05%	15	Sigma-Aldrich (493511)	-

*Previous rabbit studies used 40-100 µM, however recent work in zebrafish used a higher concentration (Nemtsas et al., 2010), which preliminary experiments demonstrated was more effective.

**Following 30 min of exposure there was a 30 min wait period in modified KH alone to allow for de-esterification of the acetoxymethyl (AM) group.

***Followed same protocol as for BAPTA-AM, with 30 min of exposure followed by a 30 min wait period in modified KH alone, but with HR measurements made after 15 min for comparison with exposure to nifedipine.

DMSO, dimethyl sulfoxide; HCN, hyperpolarisation-activated cyclic nucleotide-gated.
