# Supplementary material for: Drivers of Sinoatrial Node Automaticity in Zebrafish: Comparison With Mechanisms of Mammalian Pacemaker Function
Source: Front Physiol. 2022 Feb 28;13:818122. doi: 10.3389/fphys.2022.818122 (PMC8919049; doi:10.3389/fphys.2022.818122)
Supplement: Supplementary file 3 [file Table_3.pdf]

Supplementary Table 3. Antibodies used for immunofluorescence.

Antibody	Host	Concentration	Vendor (Catalogue)
HCN4	Rabbit	1:50	Alomone Labs (APC-052)
RyR	Mouse	1:100	Fisher Scientific (MA3-916)
AlexaFluor555 (anti-rabbit)	Goat	1:300	Fisher Scientific (A-21429)
AlexaFluor647 (anti-mouse)	Goat	1:300	Fisher Scientific (A-21236)
