# Supplementary material for: Drivers of Sinoatrial Node Automaticity in Zebrafish: Comparison With Mechanisms of Mammalian Pacemaker Function
Source: Front Physiol. 2022 Feb 28;13:818122. doi: 10.3389/fphys.2022.818122 (PMC8919049; doi:10.3389/fphys.2022.818122)
Supplement: Supplementary file 4 [file Table_4.pdf]

Supplementary Table 4. Composition of immunofluorescence solutions.

Antibody Incubation	Composition	Vendor (Catalogue)
Phosphate Buffered Saline (PBS)	0.01 M (phosphate)	Sigma-Aldrich (P3813)
NaCl	0.138 M	Sigma-Aldrich (S5886)
KCl	0.0027 M	Sigma-Aldrich (P5405)
Dimethyl Sulfoxide (DMSO)	65 mM	Fisher Scientific (BP231-1)
Triton X-100	10 µM	Sigma-Aldrich (T9284)
Bovine Serum Albumin (BSA)	0.1% w/v	Sigma-Aldrich (T9284)

CUBIC-R1	Composition	Vendor (Catalogue)
Urea	25% w/w	Fisher Scientific (U15-500)
N’,N’,N’,N’-Tetrakis	25% w/w	Sigma-Aldrich (122262)
Triton X-100	15% w/w	Sigma-Aldrich (T9284)
Type 1 H_2O	35% w/w	-
