# Supplementary material for: Drivers of Sinoatrial Node Automaticity in Zebrafish: Comparison With Mechanisms of Mammalian Pacemaker Function
Source: Front Physiol. 2022 Feb 28;13:818122. doi: 10.3389/fphys.2022.818122 (PMC8919049; doi:10.3389/fphys.2022.818122)
Supplement: Supplementary file 5 [file Table_5.pdf]

Supplementary Table 5. Effects of pharmacological block of membrane and Ca^{2+} 'clock' components on heart rate (HR) and the inter-beat variability of cycle length (measured as the root mean square of its successive differences, $RMSSD_{CL}$) in the isolated zebrafish heart.

Membrane 'Clock' Components

**Target**	**Antagonist**	**Pre-drug HR (bpm)**	**Post-drug HR (bpm)**	***p*-value**	**Pre-drug $RMSSD_{CL}$ (ms)**	**Post-drug $RMSSD_{CL}$ (ms)**	***p*-value**	***n* (Hearts)**
HCN Channels (Intracellular)	Ivabradine Hydrochloride	194 ± 13	26 ± 6*	<0.001	2.5 ± 0.5	106.3 ± 25.1*	0.006	7
HCN Channels (Extracellular)	Cesium Chloride (CsCl)	190 ± 13	50 ± 7*	<0.001	3.0 ± 0.7	43.8 ± 12.9*	0.023	6
T-type Ca^{2+} Channels	Nickel(II) Chlroide ($NiCl_2$)	178 ± 14	160 ± 13*	0.016	3.9 ± 0.7	6.1 ± 1.4	0.100	7

Ca^{2+} 'Clock' Components

**Target**	**Antagonist**	**Pre-drug HR (bpm)**	**Post-drug HR (bpm)**	***p*-value**	**Pre-drug $RMSSD_{CL}$ (ms)**	**Post-drug $RMSSD_{CL}$ (ms)**	***p*-value**	***n* (Hearts)**
Ryanodine Receptors	Ryanodine	148 ± 12	80 ± 9*	0.005	11.7 ± 3.8	54.4 ±14.2*	0.036	6
Cytosolic Ca^{2+}	BAPTA-AM	168 ± 17	125 ± 20*	0.029	4.1 ± 0.3	5.5 ± 0.9	0.198	7
L-type Ca^{2+} Channels	Nifedipine	209 ± 14	208 ± 11	0.813	2.4 ± 0.7	2.8 ± 0.6	0.626	6

Controls

**Target**	**Pre-drug HR (bpm)**	**Post-drug HR (bpm)**	***p*-value**	**Pre-drug $RMSSD_{CL}$ (ms)**	**Post-drug $RMSSD_{CL}$ (ms)**	***p*-value**	***n* (Hearts)**
Time (H_2O)	189 ± 10	197 ± 9	0.239	5.7 ± 2.4	3.5 ± 0.5	0.341	7
DMSO	172 ± 15	168 ± 15	0.300	3.8 ± 1.4	4.1 ± 1.2	0.660	6
Ethanol	187 ± 10	184 ± 11	0.354	3.3 ± 0.5	3.9 ± 0.6	0.077	8

Data is shown as mean ± standard error of the mean (SEM), and compared by paired, two-tailed, Student's T-tests; *$p<0.05$ *vs* pre-drug.

HCN, hyperpolarisation-activated cyclic nucleotide-gated.
