# Supplementary material for: Drivers of Sinoatrial Node Automaticity in Zebrafish: Comparison With Mechanisms of Mammalian Pacemaker Function
Source: Front Physiol. 2022 Feb 28;13:818122. doi: 10.3389/fphys.2022.818122 (PMC8919049; doi:10.3389/fphys.2022.818122)
Supplement: Supplementary file 6 [file Table_6.pdf]

Supplementary Table 6. Action potential (AP) characteristics from the sinoatrial node, atrium, and ventricle with and without blebbistatin.

Sinoatrial Node (SAN)

AP Parameter	Control (*n* impalements= 10)†	Blebbistatin (*n* impalements= 11)†	*p*-value *vs* Control	*p*-value *vs* Atrium	*p*-value *vs* Ventricle
Heart Rate (bpm)	140 ± 4	133 ± 8	0.463	-	-
Slope of DD (mV/s)	28 ± 4	17 ± 2*	0.028	-	-
MDP (mV)	-46 ± 3^{A,V}	-42 ± 2	0.285	0.049	0.008
dV/dt_{max} (mV/ms)	9.6 ± 1.4^{A,V}	10.1 ± 1.3	0.782	<0.001	0.020
AP Amplitude (mV)	59 ± 5^{A,V}	53 ± 3	0.295	0.001	<0.001
Max V_m	14 ± 3^{A,V}	12 ± 2	0.542	<0.001	<0.001
APD_{50} (ms)	63 ± 3^{A,V}	62 ± 3	0.799	0.005	<0.001
APD_{80} (ms)	78 ± 3^{A,V}	76 ± 3	0.711	0.032	<0.001

Atrium

AP Parameter	Control (*n* impalements= 14)†	Blebbistatin (*n* impalements= 17)†	*p*-value *vs* Control	*p*-value *vs* SAN	*p*-value *vs* Ventricle
Resting V_m (mV)	-65 ± 2^{S}	-60 ± 2	0.149	0.049	0.930
dV/dt_{max} (mV/ms)	19.6 ± 0.9^{S,V}	18.2 ± 1.0	0.329	<0.001	0.020#
AP Amplitude (mV)	92 ± 3^{S}	88 ± 5	0.475	0.001	0.925
Max V_m	28 ± 2^{S}	28 ± 2	0.891	<0.001	0.958
APD_{50} (ms)	87 ± 3^{S,V}	99 ± 3*	0.006	0.005	<0.001
APD_{80} (ms)	108 ± 2^{S,V}	117 ± 3*	0.012	0.032	<0.001

Ventricle

AP Parameter	Control (*n* impalements= 21)†	Blebbistatin (*n* impalements= 24)†	*p*-value *vs* Control	*p*-value *vs* SAN	*p*-value *vs* Atrium
Resting V_m (mV)	-66 ± 2^{S}	-64 ± 1	0.451	0.008	0.930
dV/dt_{max} (mV/ms)	16.8 ± 1.0^{S,A}	17.3 ± 0.7	0.684	0.020	0.020
AP Amplitude (mV)	94 ± 3^{S}	92 ± 3	0.585	<0.001	0.925
Max V_m	28 ± 1^{S}	27 ± 2	0.746	<0.001	0.958
APD_{50} (ms)	234 ± 4^{S,A}	221 ± 6	0.072	<0.001	<0.001
APD_{80} (ms)	271 ± 5^{S,A}	248 ± 5*	0.003	<0.001	<0.001

Data is shown as mean ± standard error of the mean (SEM), and compared by unpaired, one-way ANOVA and Tukey post hoc tests ($^{A}p<0.05$ *vs* atrium; $^{V}p<0.05$ *vs* ventricle; $^{S}p<0.05$ vs SAN) or unpaired, two-tailed, Student's T-tests (*$p<0.05$ *vs* control). †All data acquired in $N = 6$ hearts.

APD_{50} and APD_{80}, AP duration at 50% and 80% repolarisation; DD, diastolic depolarisation; dV/dt_{max}, maximum rate of change of V_m during the AP upstroke; MDP, maximum diastolic potential; V_m, membrane potential.
